# SEP enhanced the antitumor activity of 5-fluorouracil by up-regulating NKG2D/MICA and reversed immune suppression *via* inhibiting ROS and caspase-3 in mice

**DOI:** 10.18632/oncotarget.10375

**Published:** 2016-07-01

**Authors:** Mengyun Ke, Hui Wang, Yiran Zhou, Jingwen Li, Yang Liu, Min Zhang, Jie Dou, Tao Xi, Baiyong Shen, Changlin Zhou

**Affiliations:** ^1^ State Key Laboratory of Natural Medicines, School of Life Science and Technology, China Pharmaceutical University, Nanjing, Jiangsu, 210009, PR China; ^2^ Department of General Surgery, Rui Jin Hospital, Research Institute of Pancreatic Diseases, School of Medicine, Shanghai JiaoTong University, Shanghai, 200025, PR China; ^3^ Research Institute of Advanced Surgical Techniques and Engineering of Xi'an Jiaotong University, Regenerative Medicine and Surgery Engineering Research Center of Shaanxi Province, First Affiliated Hospital, Xi'an Jiaotong University, Shaanxi, Xi'an, 710061, PR China

**Keywords:** SEP, 5-fluorouracil, antitumor activity, immunotherapy, apoptosis

## Abstract

Chemotherapy and immunotherapy are the main remedies used in cancer treatment. Because immunotherapy can not only reduce the toxicity of chemotherapeutics but also enhance antitumor effects *in vivo*, combining these two therapies is a trend that continues to gain more attention in clinic. SEP, a polysaccharide isolated from *Strongylocentrotus nudus* egg, has been reported to display antitumor activity by stimulating immune cells, including NK and T cells, *via* TLR2 and TLR4. In the present study, the synergistic effect between SEP and 5-fluorouracil (5-FU), a traditional cytotoxic drug, *in vitro* and *in vivo* was investigated. The results obtained indicated that SEP alone stimulated NK-92 cytotoxicity and coordinated with 5-FU to augment the cytotoxicity of NK-92 cells against HepG-2 or A549 cells *in vitro*. SEP promoted NK-92 activity by stimulating NKG2D and its downstream DAP10/PI3K/Erk signaling pathway. Additionally, 5-FU could increase MICA expression on HepG-2 or A549 cells and prevent membrane MICA from shedding as soluble MICA, which were abrogated in the tumor cells transfected with ADAM 10 overexpression plasmid. Moreover, in H22- or Lewis lung cancer (LLC)-bearing mouse models, SEP reversed 5-FU-induced atrophy and apoptosis in both the spleen and bone marrow *in vivo* by suppressing ROS generation and caspase-3 activation. All of these results highlight the potential for the combination of SEP and 5-FU in cancer therapy in the future.

## INTRODUCTION

Cytotoxic chemotherapy is an effective treatment for cancer. Unfortunately, using cytotoxic agents not only exhibited toxicities towards normal tissues, including the development of hematopoietic and immune suppression, but also led to the occurrence of drug-resistant tumor cells [1, 2]. Recently, the combination of chemotherapy and immunotherapy has received more attention and has increasingly been used in clinical practice [3]. On one hand, on the premise of maintaining the antitumor effect of chemotherapy drugs, immunotherapy can weaken chemotherapy-induced immune injury [4]. On the other hand, based on the immune effects of chemotherapy drugs, synergy between chemotherapy and immunotherapy has indeed been seen in several clinical trails; for example, an obvious outcome of gemcitabine combined with IL-2 treatment was observed in patients with metastatic colorectal cancer [5, 6]. In these studies, chemotherapy was found to be immunomodulatory, enhance the cross-presentation of tumor antigens, and make cytotoxic CTL/NK cells more susceptible to tumor cells despite their immunosuppressive effects [5–7].

*Strongylocentrotus nudus* eggs polysaccharide (SEP) is an immunostimulating agent. Our previous research has revealed that SEP has a protective effect on cyclophosphamide (Cy)-treated mice against myelosuppression and inhibits tumor growth *in vivo* by activating the immune system, including stimulating the cytotoxicity of T cells and NK cells [8–10]. However, the synergistic effect between SEP and chemotherapy has not yet been investigated. 5-fluorouracil (5-FU), a general antimetabolite chemotherapy drug, is widely used in treatment of cancer in the clinic and exhibits suppressive effects on the immune system, including leukopenia and atrophy of hematopoietic organs [11–13]. Moreover, 5-FU is immunomodulatory and has been combined with cytokines in the clinic [14]. An adjuvant combination therapy approach including 5-FU and IFN-α has achieved an overall 5-year survival of 55% in a phase II clinical trial of pancreatic adenocarcinoma [14]. Therefore, the effect of SEP and 5-FU was further investigated in the present study.

The combined antitumor activity of 5-FU and SEP *in vitro* and *in vivo* was studied. *In vitro*, SEP directly stimulated NK-92 cytotoxicity through the upregulation of NKG2D expression and activation of the downstream DAP10/PI3K/Erk signaling pathway. Moreover, 5-FU increased and maintained membrane MICA (the ligand of NKG2D) expression on tumor cells through the inhibition of ADAM10. As a result, synergy of SEP and 5-FU enhanced the cytotoxicity of NK-92 cells toward HepG-2 or A549 cells *in vitro.* Furthermore, in H22- or Lewis Lung Cancer (LLC)-bearing mouse models, SEP not only enhanced the antitumor activity of 5-FU (medium dose) but also reversed the 5-FU-induced apoptosis of cells from both the bone marrow and spleen by reducing reactive oxygen species (ROS) generation and caspase-3 activation. These results indicated that the combined therapy of SEP and 5-FU could be a potential strategy for cancer treatment in the future.

## RESULTS

### SEP directly stimulates the activity of NK-92 cells and, coordinated with 5-FU, augments the cytotoxic effect of NK-92 cells against tumor cells

**Figure 1:**
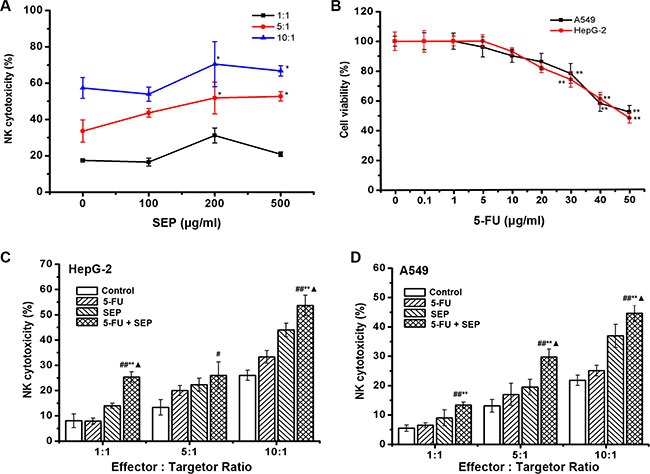
SEP promotes the cytotoxicity of NK-92 cells and, coordinated with 5-FU, enhances the cytolytic effect of NK-92 cells against HepG-2 and A549 cells (**A**) SEP up-regulated NK-92 cell cytotoxicity against K562 cells in a dose- and ratio-dependent manner. *n* = 3, The data were shown as the means ± SD, ^*^ *P* < 0.05 compared with the untreated group; (**B**) The cytotoxicity of 5-FU on HepG-2 or A549 cells were measured by an MTT assay. The data represent the means ± SD of three independent experiments. ^**^ *P* < 0.01 compared with the untreated group; (**C**–**D**) SEP combined with 5-FU stimulated NK-92 cytotoxicity against HepG-2 (C) and A549 (D) cells. NK-92 cells were treated with SEP (200 μg/mL) for 24 h, and the HepG-2 and A549 cells were treated with 5-FU (20 μg/mL) for 24 h before initiation of the cytolytic assay. The data represent the means ± SD of three independent experiments. ^#^ *P* < 0.05 and ^##^ *P* < 0.01 compared with the untreated group; ^*^ *P* < 0.05 and ^**^ *P* < 0.01 compared with the 5-FU (20 μg/mL)-treated group; ^▲^ *P* < 0.05, compared with the SEP (200 μg/mL)-treated group.

The effect of SEP on the activity of NK-92 cells was measured first. As shown in Figure 1A, the specific lysis of K562 cells was enhanced in the SEP-treated groups, indicating that SEP could directly stimulate NK cell cytotoxicity *in vitro*. We then measured the potential of SEP and 5-FU to augment the cytotoxicity of NK-92 cells against HepG-2 or A549 cells. The results of Figure 1B indicated that the 50% inhibitory concentration (IC_50_) of 5-FU on both HepG-2 and A549 cells at 24 h was approximately 50 μg/mL. Therefore, 20 μg/mL of 5-FU, approximately half of the IC_50_ value, was used in the following experiment. Co-culture of NK-92 cells pretreated with SEP (200 μg/mL, 24 h) and HepG-2 or A549 cells pretreated with 5-FU (20 μg/mL, 24 h) resulted in significantly enhanced NK cytotoxicity against both cancer cell lines (Figure 1C and 1D). Compared with the control groups, the NK lysis rates of HepG-2 cells in the SEP and 5-FU treated group was increased by 17.32%, 20.53%, and 27.73% at E:T (effector and target) ratios of 1:1, 5:1 and 10:1, respectively. Similar results were obtained in A549 cells with NK cytotoxicity increased to 13.42%, 29.83%, and 44.75% at E:T ratios of 1:1, 5:1 and 10:1, respectively (Figure 1D).

### SEP activates NK-92 cells through the NKG2D pathway

**Figure 2:**
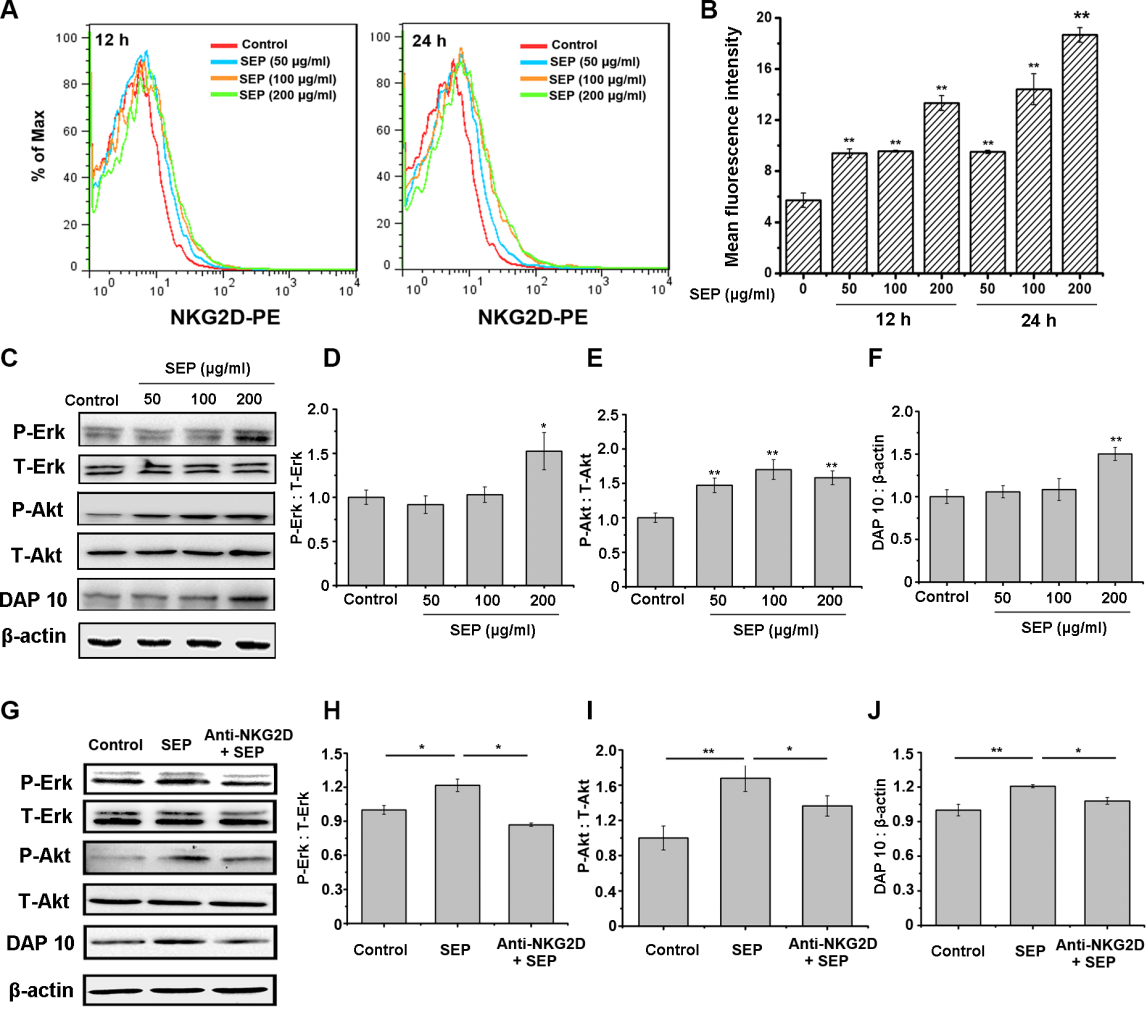
SEP-stimulated NK-92 cells are mediated by NKG2D activation (**A**–**B**) SEP up-regulates the NKG2D expression on NK-92 cells in a dose- and time-dependent manner. The mean fluorescence intensity in each group is examined using flow cytometry. The histogram overlays shown in panel A are representative of triplicates, and the combined results are presented in panels B. (**C**–**F**) SEP activates the expression of DAP10 and the phosphorylation of Akt and Erk in NK-92 cells after 24 h. Densitometric analysis of Erk (D) and the phosphorylation of Akt (E) and DAP10 (F) protein levels in Western blots of NK-92 cells (C). Data are shown as the means ± SD from three separate experiments. ^*^ *P* < 0.05 and ^**^ *P* < 0.01 compared with the control group. (**G**–**J**) Blocking NKG2D with neutralizing antibody attenuates SEP-activated DAP10 and the phosphorylation of Akt and Erk induced by SEP in NK-92 cells after 24 h. Densitometric analysis of Erk (H), the phosphorylation of Akt (I), and DAP10 (J) protein levels in western blots of NK-92 cells (G). Data are shown as the means ± SD from three separate experiments. ^*^ *P* < 0.05 and ^**^ *P* < 0.01 compared with the SEP-treated group. Representative blots are shown from three independent experiments.

Recent studies have shown that NKG2D is one of the most important stimulatory receptors expressed on NK cells, and phosphatidylinositol-3 kinase (PI3K) is the most common signaling mediator downstream of NKG2D [15]. Furthermore, the PI3K-ERK pathway has been shown to play an important role in NK cell activation and cytotoxicity [16, 17]. Thus, we hypothesized that SEP first promotes the expression of NKG2D receptors and then activates the downstream DAP10/PI3K/Erk pathway, leading to the activation of NK cells. To confirm this hypothesis, we first examined if activation of NK cells by SEP was mediated by NKG2D. As presented in Figure 2A, 2B, NKG2D expression on NK-92 cells was up-regulated in the SEP-treated groups.

Additionally, the activation of DAP10 and phosphorylation of Akt and Erk were observed in the SEP-treated NK-92 cells (Figure 2C–2F), which were impaired by the NK-92 cells pretreated with NKG2D neutralizing antibody (Figure 2G–2J). Specifically, when the cells pretreated with anti-NKG2D and further inoculated with SEP, the level of DAP10 and the phosphorylation of PI3K and ERK were decreased by 18.2%, 31.1% and 35.4%, respectively, compared with the cells treated with isotype antibody and SEP. The results mentioned above suggested that SEP facilitated the function of NK cells *via* the NKG2D/DAP10/PI3K/Erk pathway.

### 5-FU up-regulates and maintains MICA expression on tumor cells

**Figure 3:**
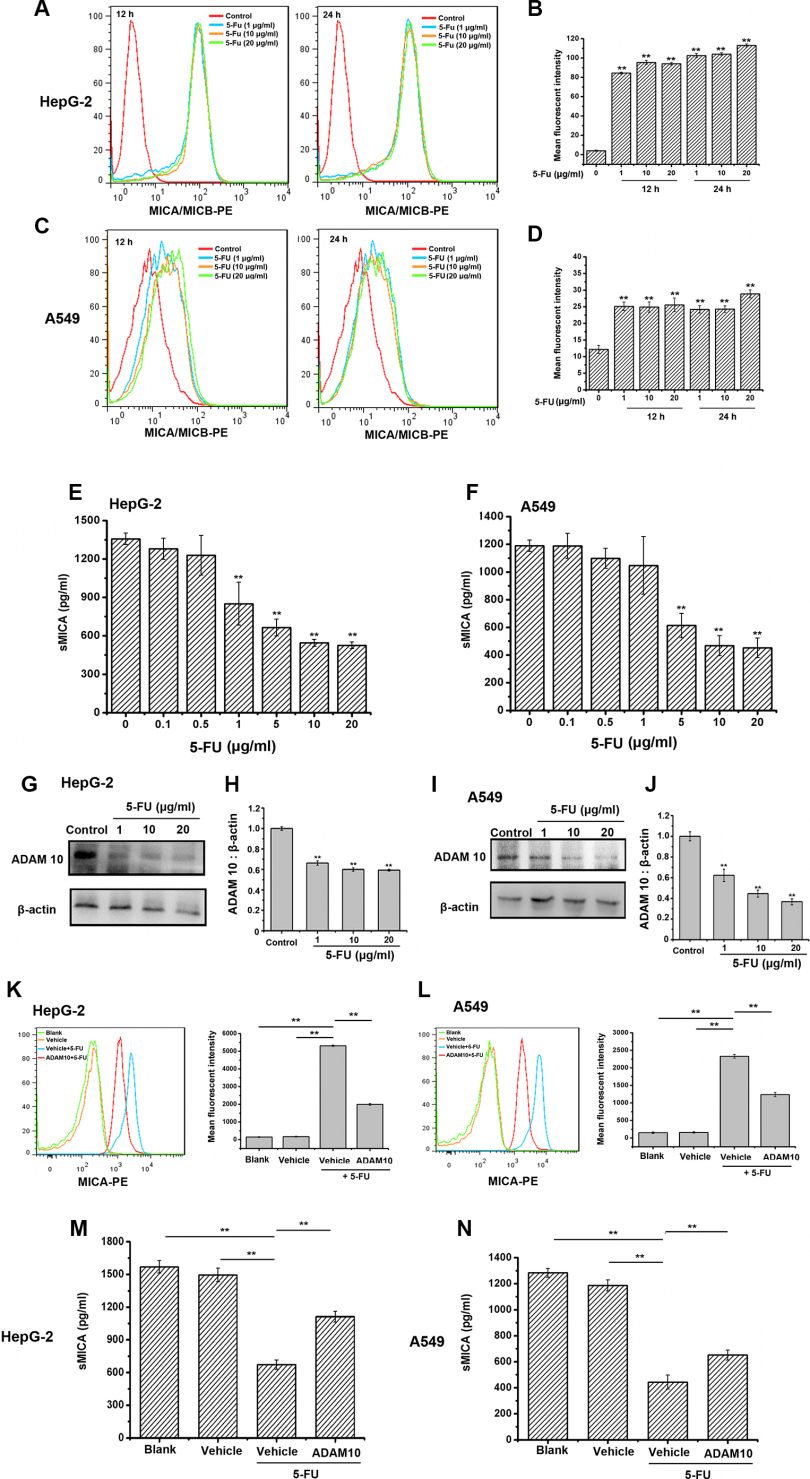
5-FU enhances and maintains the expression of membrane MICA on HepG-2 and A549 cells by preventing ADAM10 expression (**A**–**D**) The membrane MICA expression on HepG-2 and A549 cells is enhanced after being exposed to 20 μg/mL of 5-FU for 24 h. The mean fluorescence intensity in each group is examined using flow cytometry. The histogram overlays shown in panels A and C are representative of triplicates, and the combined results are presented in panels B and D. (**E**–**F**) 5-FU inhibits sMICA secretion in a dose-dependent manner in the supernatant of HepG-2 (E) and A549 (F) cells after 24 h. (**G**, **I**) 5-FU suppresses the protein expression of ADAM10 in HepG-2 (G) and A549 (I) cells in a dose-dependent manner. H, J. Densitometric analysis of ADAM10 protein levels in Western blots of HepG-2 (**H**) and A549 cells (**J**). Values are presented as the means ± SD from three separate experiments. ^**^ *P* < 0.01 compared with the control group. Shown are representative blots from three independent experiments with similar results. (**K**–**L**) The increasement of membrane MICA expression on HepG-2 (K) or A549 cells (L) induced by 5-FU is impaired by the overexpression of ADAM10. Tumor cells were divided into ADAM10 group (ADAM10-overexpressed plasmid group), vehicle group (blank-Vector-transfected group) and blank group (no DNA-transfected group), and then incubated with 5-FU (20 μg/mL) for further 24 h. The mean fluorescence intensity in each group is analyzed using flow cytometry. The histogram overlays shown in left panels are the representative of triplicates, and the combined results are presented in right panels. (**M**–**N**) ADAM 10 overexpression attenuates 5-FU-suppressed sMICA secretion in the supernatant of HepG-2 (M) and A549 (N) cells was weaken in the ADAM 10 overexpression group. Data are presented as the means ± SD from three separate experiments. ^**^ *P* < 0.01 compared with the cells in the vehicle group treated with 5-FU.

It is well known that MICA and MICB, which are the ligands of NKG2D and expressed on tumor cells, are important molecules for stimulating the cytoxicity of immune cells, and MICA can easily fall off to soluble MICA (sMICA), which inhibits the activity of NK cells [18, 19]. Therefore, the effect of 5-FU on MICA expression of tumor cells was studied further. 5-FU increased and maintained membrane MICA/MICB expression on HepG-2 and A549 cells within 24 h (Figure 3A–3D). Additionally, sMICA in the culture supernatants of HepG-2 and A549 cells treated with 5-FU for 24 h were decreased in a dose-dependent manner (Figure 3E, 3F). Meanwhile, 5-FU suppressed the expression of ADAM 10, which promoted MICA shedding, in both HepG-2 and A549 cells (Figure 3G–3J). Moreover, when ADAM 10 was overexpressed in these tumor cells, the 5-FU-induced the upregulation of MICA expression and downregulation of sMICA secretion were both attenuated (Figure 3K–3N). Compared with the cells in the vehicle group treated with 5-FU, the sMICA secretion was increased by 65.4% and 46.9% in the HepG-2 and A549 cells transfected in ADAM 10 overexpression plasmid treated with 5-FU, respectively. Therefore, the inhibition of ADAM10 induced by 5-FU could one of the mechanisms utilized for inducing and maintaining MICA expression on tumor cells.

### SEP enhances the antitumor activity of 5-FU in H22- or LLC-bearing mice

**Figure 4:**
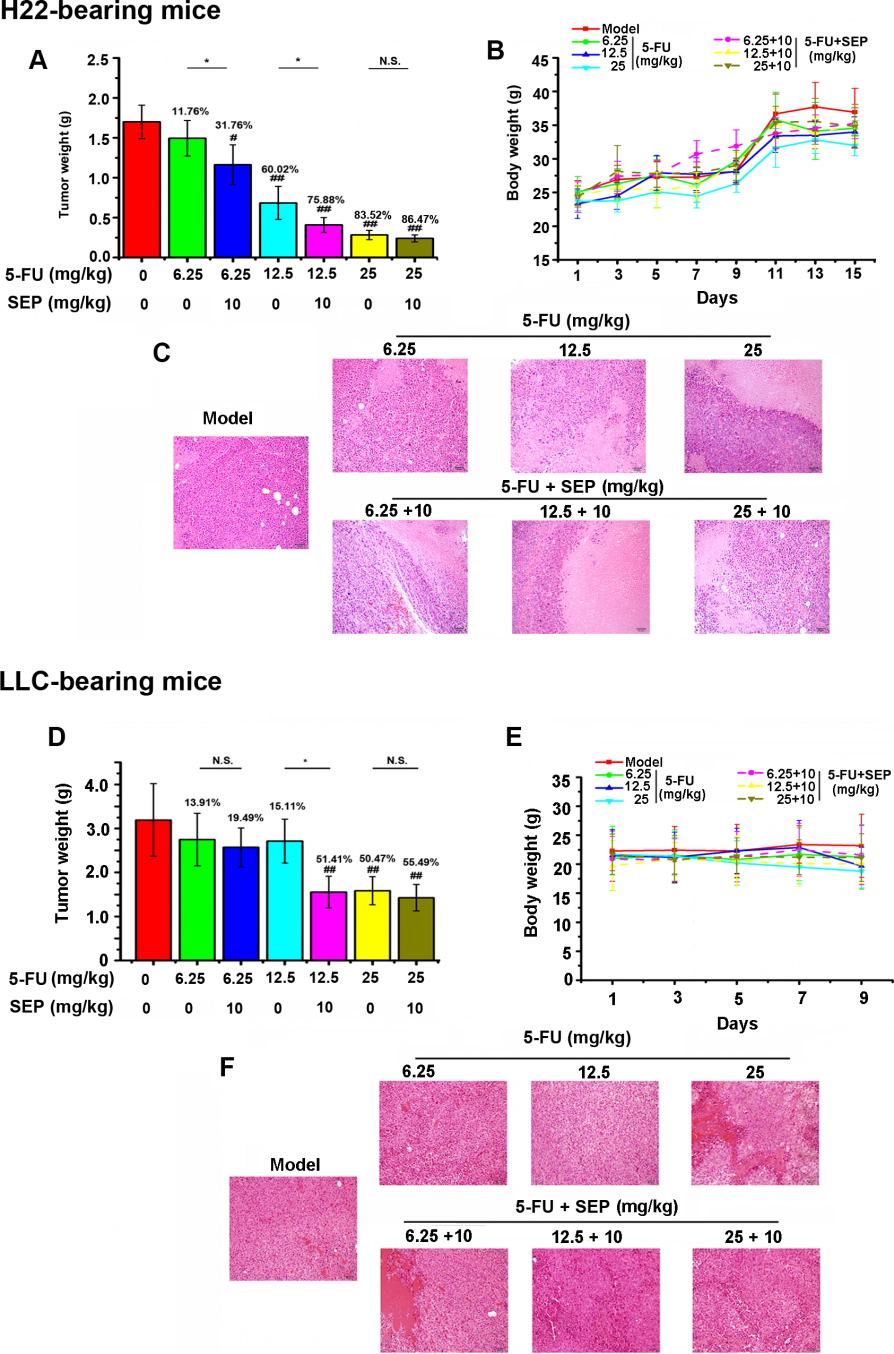
The antitumor effect of 5-FU is enhanced after being combined with SEP in H22- or LLC-bearing mice (**A**, **D**) The tumor weights in H22- or LLC-bearing mouse models are significantly inhibited in the 5-FU combined with SEP treatment group. (**B**, **E**) Animal weight curves in H22- or LLC-bearing mouse models during the drug administration. (**C**, **F**) Pathological changes in H22- or LLC-bearing mice. Each group contains 12 mice. Data are presented as the means ± SD. ^#^ *P* < 0.05 and ^##^ *P* < 0.01 compared with the control group. ^*^ *P* < 0.05, ^**^ *P* < 0.01 and N.S., short for no significance, compared with the corresponding dose of 5-FU.

Because SEP and 5-FU treatment synergistically stimulates NK activity to kill tumor cells *in vitro*, the antitumor effect of the combination therapy was further studied *in vivo* by establishing H22- or LLC-bearing mouse models. Compared with 5-FU treatment alone, co-treatment of SEP and 5-FU significantly suppressed tumor growth in these two models (Figure 4A, 4D). As presented in Figure 2A, in H22-bearing mice, tumor weights in the combined therapy group were obviously decreased relative to the 6.25 or 12.5 mg/kg 5-FU (quarter or half of the highest dose) alone treated group. Specifically, the tumor inhibitory rate reached 75.88% in the 5-FU (12.5 mg/kg) combined with SEP (10 mg/kg) group (Figure 4A). Similar results were obtained in LLC-bearing mice. The tumor inhibitory rate in the 5-FU combined with SEP 12.5 + 10 mg/kg group was increased by 36.30% compared with the 12.5 mg/kg 5-FU group (Figure 4D) and was the same as that of the 25 mg/kg 5-FU group. Moreover, as shown in Figure 4C and 4F, H&E staining of tumor tissues in each group shows that the necrotic regions in the combination group (5-FU + SEP) were larger than those in the 5-FU-alone treated group.

The curves demonstrated that the body weights were obviously decreased as the dosage of 5-FU increased in both tumor-bearing models, whereas in the combined therapy of 5-FU and SEP, the body weight losses were reduced (Figure 4B, 4E). The results described above suggest that, in combination therapy-treated H22- or LLC-bearing mice, SEP not only improved the antitumor activity of 5-FU but also reduced the body loss induced by 5-FU.

### SEP protects the thymus and spleen from atrophy induced by 5-FU *in vivo*

**Figure 5:**
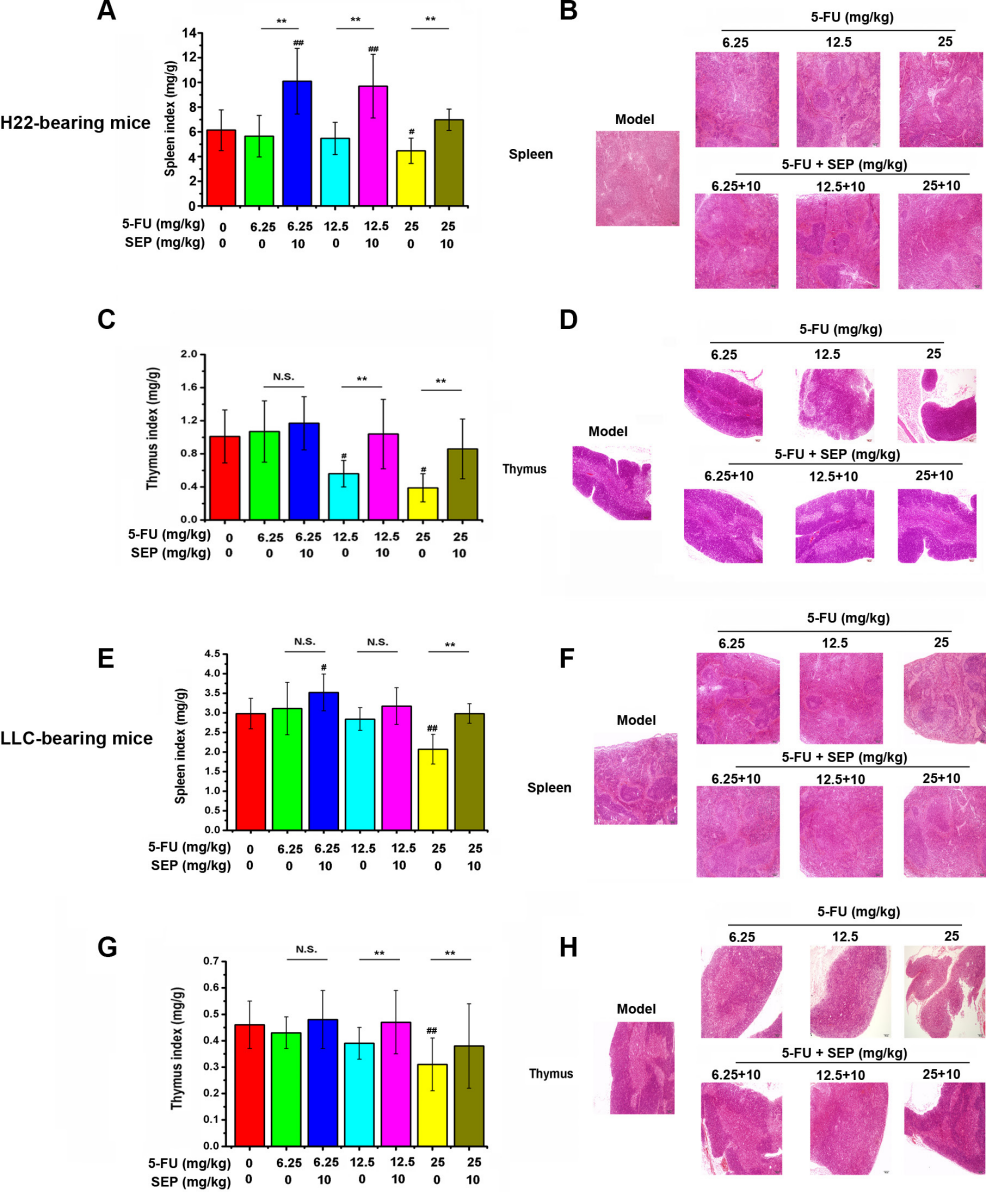
Atrophy of the thymus and spleen caused by 5-FU is attenuated by SEP *in vivo* (**A**, **C**, **E**, **G**) SEP up-regulates the indices of the thymus and spleen, which are decreased by 5-FU in H22- or LLC-bearing mice. (**B**, **D**, **F**, **H**) Hematoxylin-eosin (H&E) staining of the thymus and spleen present the atrophy and lesions of these two tissues in the SEP and 5-FU administered groups, which were alleviated compared with 5-FU alone treated group. Representative micrographs are shown (100×, H&E stain). *N* = 12; values are presented as the means ± SD. ^##^ *P* < 0.01 compared with the control group. ^*^ *P* < 0.05, ^**^ *P* < 0.01, and N.S. (short for no significance) compared with the corresponding dose of the 5-FU group.

To evaluate the effects of combination therapy on immune organs, we examined changes in the spleen and thymus in these two models. 5-FU-induced decreases in spleen and thymus indices, and these indices were both elevated by 10 mg/kg SEP (Figure 5A, 5C, 5E and 5G). In H22-bearing mice, the spleen and thymus indices in the 5-FU combined with SEP group (25 + 10 mg/kg) were elevated 1.58- and 2.21-fold compared to those of the 25 mg/kg 5-FU group, respectively (Figure 5A, 5C). Similar results were observed in the LLC-bearing mice, the spleen and thymus indices in the 5-FU combined with SEP were both up-regulated and were similar to those in the model group (Figure 5E, 5G). In H&E staining of the spleens and thymus tissues shown, compared with the 5-FU group, the atrophy degrees in SEP combined with the 5-FU-treated groups were alleviated (Figure 5B, 5D, 5F, and 5H). These results suggested that SEP could reverse the atrophy of immune organs induced by 5-FU *in vivo*.

### SEP impairs 5-FU-induced suppression of hematopoietic functions in bone marrow *in vivo*

**Figure 6:**
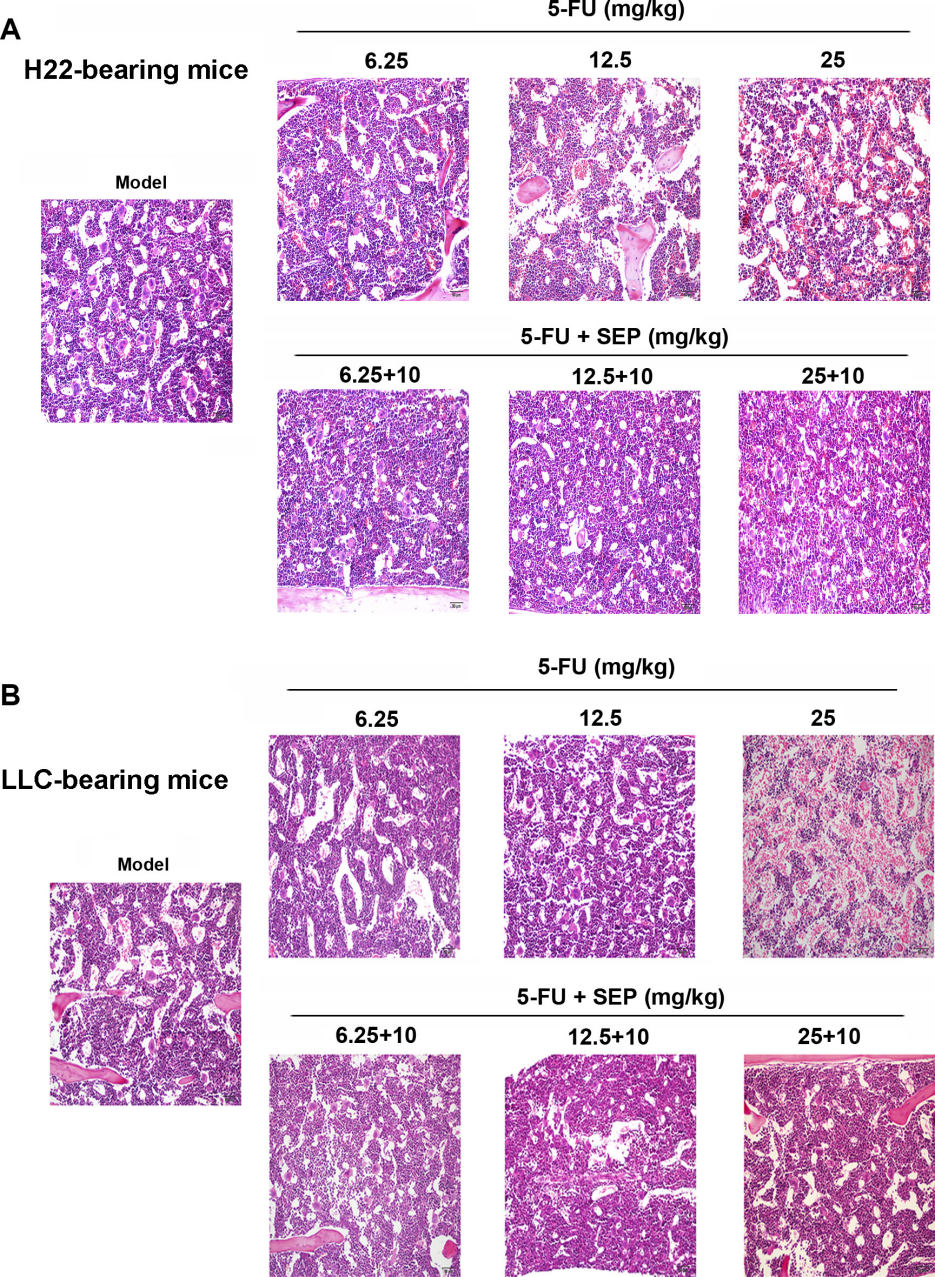
Inhibition of hematopoietic functions in bone marrow caused by 5-FU are restored in the SEP combined treatment group *in vivo* The extent of hematopoietic suppression in SEP and 5-FU treated groups are lighter than that in the 5-FU alone treated group in both H22-(**A**) or LLC-bearing mice models (**B**). Representative micrographs are shown (100×, H&E stain).

To evaluate the hematopoietic function of the bone marrow, we counted the leukocytes, erythrocytes, reticulocytes and platelets from the peripheral blood and CD34^+^ cells from the bone marrow in each group. As shown in Tables 1 and 2, in both tumor-bearing mouse models, the numbers of leukocytes, erythrocytes, reticulocytes and platelets as well as the percentage of CD34^+^ cells were elevated remarkably in combined therapy of SEP and 5-FU groups compared to 5-FU-treated group. In H22-bearing mouse models at the high dose of 25 mg/kg of 5-FU, the number of hematopoietic function-related cells were significantly decreased, and these cells were recovered to numbers similar to those in the model group after treatment with both 25 mg/kg 5-FU and10 mg/kg SEP (Table 1). Additionally, in LLC-bearing mouse models, the leukocytes, erythrocytes, reticulocytes, platelets and CD34^+^ cells in the 5-FU and SEP combined group (25 + 10 mg/kg) were 3.43-, 1.44, 1.60-, 9.00- and 1.67-fold compared to the 25 mg/kg 5-FU group (Table 2). H&E staining of femurs indicated that the decrease of hematopoietic cells in each co-treated group was attenuated compared to the relative 5-FU-alone treated group (Figure 6), suggesting that SEP exhibited a protective effect on the hematopoietic function of bone marrow inhibited by 5-FU.

**Table 1:** Influence of SEP combined 5-Fu treatment on haemopoietic function in H22-bearing mice

Group	Dosage (mg/kg)	WBC (×10^9^)	RBC (×10^12^)	PLT(×10^9^)	RC (×10^12^)	CD34^+^ (%)
Model		5.02 ± 0.18	7.19 ± 0.22	240.34 ± 69.23	0.38 ± 0.03	2.08 ± 0.14
5-FU	6.25	4.6 ± 0.06	7.17 ± 0.18	173.56 ± 48.22	0.36 ± 0.01	1.61 ± 0.08^##^
	12.5	3.48 ± 0.12^##^	6.15 ± 0.11	160.65 ± 37.34^#^	0.32 ± 0.01	1.31 ± 0.09^##^
	25	2.42 ± 0.22^##^	5.80 ± 0.23^#^	115.89 ± 29.11^##^	0.19 ± 0.01^##^	0.99 ± 0.02^##^
5-FU + SEP	6.25 + 10	4.75 ± 0.17	7.23 ± 0.08	212.52 ± 56.22	0.41 ± 0.01	1.81 ± 0.12
	12.5 + 10	4.02 ± 0.09^**^	6.78 ± 0.22	200.36 ± 40.48^**^	0.38 ± 0.02	1.50 ± 0.09^##^^*^
	25 + 10	3.74 ± 0.14^#^^**^	6.53 ± 0.26^**^	180.39 ± 37.63^#^^**^	0.34 ± 0.01^**^	1.16 ± 0.07^##^^**^

The data were shown as mean ± SD,

# *P* < 0.05,

## *P* < 0.01, compared with model group.

* *P* < 0.05,

** *P* < 0.01, compared with the corresponding dose of the 5-FU group.

**Table 2:** Influence of SEP combined 5-Fu treatment on haemopoietic function in LLC-bearing mice

Group	Dosage (mg/kg)	WBC (×10^9^)	RBC (×10^12^)	PLT(×10^9^)	RC (×10^12^)	CD34^+^ (%)
Model		7.14 ± 1.23	5.68 ± 1.35	280.09 ± 66.81	0.67 ± 0.25	2.09 ± 0.12
5-FU	6.25	4.45 ± 1.60	5.96 ± 1.66	262.67 ± 54.98	0.27 ± 0.03^##^	1.94 ± 0.07
	12.5	3.48 ± 0.73^##^	5.68 ± 1.35	214.86 ± 45.96^#^	0.18 ± 0.01^##^	1.15 ± 0.07^##^
	25	1.41 ± 0.15^##^	3.71 ± 1.17^##^	142.00 ± 25.09^##^	0.04 ± 0.01^##^	0.92±0.08^##^
5-FU + SEP	6.25 + 10	5.43 ± 0.63	6.61 ± 0.81	282.67 ± 71.97	0.48 ± 0.15^**^	1.85 ± 0.09
	12.5 + 10	6.49 ± 1.61^**^	6.07 ± 1.57	241.33 ± 46.46	0.57 ± 0.14^**^	1.69 ± 0.17^#^^**^
	25 + 10	4.84 ± 0.23^##^^**^	5.33 ± 0.26^**^	227.39 ± 48.78^**^	0.36 ± 0.12^##^^**^	1.54 ± 0.08^#^^**^

The data were shown as mean ± SD,

# *P* < 0.05,

## *P* < 0.01, compared with model group.

* *P* < 0.05,

** *P* < 0.01, compared with the corresponding dose of the 5-FU group.

### SEP prevents 5-FU-induced apoptosis in splenocytes and bone marrow cells *in vivo*

**Figure 7:**
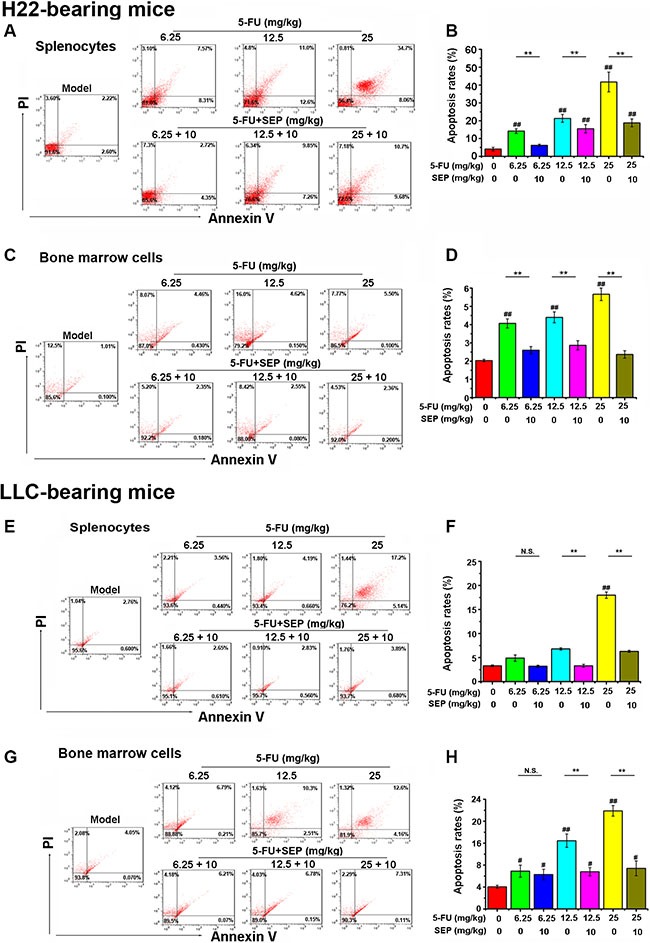
5-FU-induced apoptosis in splenocytes and bone marrow cells are impaired *in vivo* Cell apoptosis analysis in splenocytes and bone marrow cells is conducted by flow cytometry using AV/PI staining. (**A**, **C**, **E**, **G**) Late apoptotic cells that bind AV and PI are in the upper right quadrant, whereas early apoptotic cells binding AV are in the lower right quadrant. Representative dot plots show the distribution of different cell populations. (**B**, **D**, **F**, **H**) Percentage of apoptosis. Respective data from at least three independent experiments are summarized and presented as the means ± SD. ^*^ *P* < 0.05, ^**^ *P* < 0.01 and N.S. (short for no significance) compared with the corresponding dose of the 5-FU group.

As shown in Figure 7, 5-FU can induce the apoptosis of splenocytes and bone marrow cells in H22- or LLC-bearing mouse models, but after treatment combined with SEP, the apoptosis of these cells was significantly inhibited.

**Figure 8:**
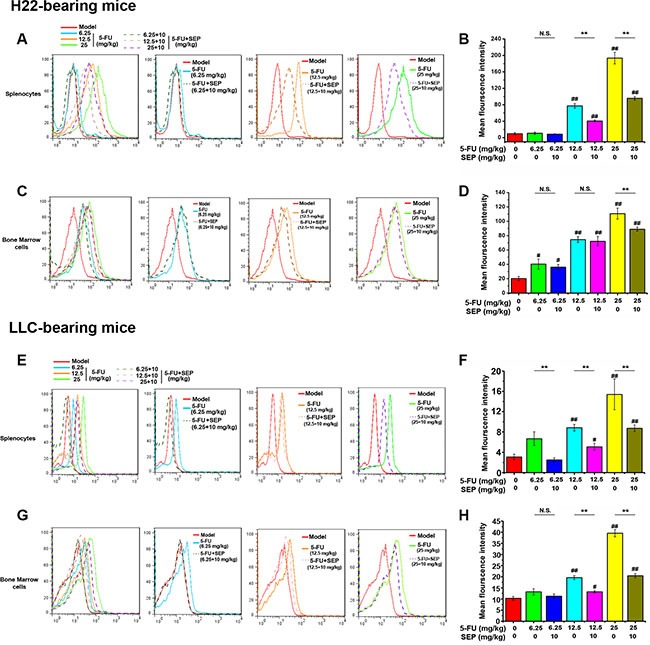
ROS generation induced by 5-FU is inhibited by SEP in splenocytes and bone marrow cells *in vivo* Splenocytes and bone marrow cells in each group are subjected to flow cytometric analysis for ROS using the fluorescent probe DCFH-DA. The histogram overlays shown in panels (**A**, **C**, **E**, and **G**) are representative of triplicates, and the combined results are presented in panels (**B**, **D**, **F**, and **H**) Respective data from at least three independent experiments are summarized and presented as the means ± SD. ^*^ *P* < 0.05, ^**^ *P* < 0.01, and N.S. (short for no significance) compared with the corresponding dose of the 5-FU group.

**Figure 9:**
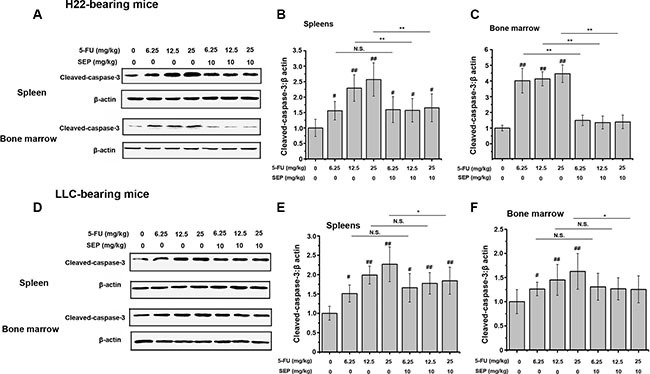
5-FU-activated caspase-3 is prevented in splenocytes and bone marrow cells *in vivo* (**A**) Proteins of splenocytes and bone marrow cells in each group are subjected to Western blot analysis. 5-FU-activates caspase-3 expression in splenocytes and bone marrow cells, which is suppressed in the SEP and 5-FU treatment groups. Densitometric analysis of cleaved caspase-3 protein levels in Western blots. Data are shown as the means ± SD from three separate experiments. ^*^ *P* < 0.05 and ^**^ *P* < 0.01 compared with the control group. Shown are representative blots from three independent experiments with similar results.

Since ROS generation and caspase-3 activation are vital factors in 5-FU-induced apoptotic effects, both ROS secretion and caspase-3 expression in the spleen and bone marrow were detected further. In H22- or LLC-bearing mouse models, 5-FU stimulated both ROS secretion and cleaved caspase-3 expression of splenocytes and bone marrow cells *in vivo* (Figures 8 and 9). Whereas in the combination group of SEP combined with every dose of 5-FU, the expression levels of both ROS and cleaved caspase-3 were significantly decreased (Figures 8 and 9).

## DISCUSSION

A combinatory approach of using both chemotherapy and immuno-therapy is a research hotspot for treating cancer in the clinic because immunotherapy can reduce the immune damage induced by chemotherapy, and the combination of chemo- and immunotherapy exhibits a synergistic antitumor effect [20, 21]. Our previous research has found that SEP inhibits tumor growth *in vivo* by stimulating immune cells, including T and NK cells [8–10]. Additionally, 5-FU is a widely used chemotherapy agent for cancer therapy in the clinic. Despite the immunosuppressive effects of 5-FU, several research studies have investigated the effect of 5-FU combined with an immunoregulatory agent, such as IL-12 and IL-15 [21, 22]. In this study, the effect of the combination therapy of SEP and 5-FU on antitumor activity and immune and hematopoietic systems were investigated *in vitro* and *in vivo*.

As is known, NK cells are important components of the innate immune response and contribute substantially to the antitumor immune response [23]. As our previous research reported [8], SEP not only inhibited tumor growth, but also promoted NK-mediated cytotoxicity and enhanced the NK1.1^+^ cell population in LLC-bearing mice. Moreover, SEP significantly suppressed human NSCLC H460 growth in nude mice, which was abrogated by the selective depletion of NK cells *via* the intraperitoneal injection of anti-asialo GM-1 antibodies. Therefore, SEP-enhanced NK cytotoxicity is crucial and that NK cells is a unique SEP target in tumor-bearing mice. In this study, the data obtained *in vitro* indicated that SEP could directly enhance the cytotoxicity of NK-92 cells against K-562 cells. It has been reported that NK cell activity is controlled by a balance of signals derived from inhibitory and activating receptors, and one of the most powerful activating receptors expressed by NK cells is NKG2D, which can recognize stress-induced ligands that appear on the surface of tumor cells [24, 25]. NKG2D associates with the adaptor protein DAP10, which contains a YINM-sequence signaling motif [26, 27]. After receptor stimulation, the tyrosine is phosphorylated, and PI3K and a Grb2-Vav1 complex were consequently activated, leading to the downstream activation of Akt and MAP kinases, respectively [26, 28]. In this study, the expression of NKG2D on NK-92 cells was up-regulated in the SEP-treated group. Moreover, the increased DAP10 activation and Akt and Erk phosphorylation were also observed after SEP treatment and these phenomena were attenuated after anti-NKG2D treatment. Thus, SEP might first increase the NKG2D/DAP10 complex in NK-92 cells and then stimulate the downstream phosphorylation of Akt and Erk and thereby activate NK cytotoxicity.

NKG2D ligands mainly include MHC class I chain-related A and B (MICA/B) molecules and UL16-binding proteins (ULBPs) 1–6 [29–31]. In contrast to classic MHC class I molecules, MICA/B are rarely expressed on normal cells but are frequently expressed on tumor cells [32–34]. The engagement of MICA/B and NKG2D strongly activates NK cells and co-stimulates T cells, enhancing their cytolytic activity and cytokine production [35]. Thus, the MICA/B-NKG2D pathway is an important mechanism by which the host immune system recognizes and kills transformed cells [36]. In addition to those membrane-bound forms, MICA/B molecules are also cleaved proteolytically from tumor cells and appear as soluble forms in the sera of patients with malignancies [37–39]. Soluble MICA/B in circulation down-regulates NKG2D expression and disturbs NKG2D-mediated antitumor immunity, and as a result, tumor cells escape from NKG2D-mediated immuno-surveillance [40]. Additionally, ADAM10 is involved in the switch between membrane-bound and soluble MICA. ADAM10 knockdown resulted in increased expression of membrane-bound MICA, decreased production of soluble MICA, and enhanced NK sensitivity of tumor cells [34, 41]. It has been reported 5-FU could regulate the MICA expression on A549 cells [42]. Similarly, we are also delighted to find out that 5-FU not only induced membrane MICA expression on HepG-2 and A549 cells, but also inhibited MICA shedding as sMICA, which was mediated by inhibiting the expression of ADAM10, and that SEP promoted the expression of NKG2D on NK-92 cells and then activated the downstream DAP10/PI3K/Erk pathway, further enhancing the NKG2D-mediated antitumor immunity of NK cells. Therefore, the coordinative interaction of SEP and 5-FU on the enhancement of NK activity against HepG-2 or A549 cells is based on triggering the interaction between NKG2D and MICA.

Considering the combined therapeutic effect of 5-FU and SEP *in vitro*, the antitumor activity of these two drugs is further studied *in vivo* by establishing H22- or LLC-bearing mouse models. The data obtained indicated that SEP remarkably enhanced the antitumor activity of 5-FU. In 5-FU (12.5 mg/kg, half of the high dose) combined with SEP (10 mg/kg) group, tumor weights were significantly suppressed, and the tumor inhibitory rates were obviously elevated and similar to those in the group treated with 5-FU at high dose (25 mg/kg). Additionally, body loss in the combination therapy group was remarkably alleviated compared with that in the 5-FU alone treated group, indicating that SEP could reduce the toxicity of 5-FU *in vivo*.

Immune suppression and atrophy of hematopoietic organs are the two dominant toxicities related to 5-FU applications in humans and mice [11–13]. Myelosuppression, as a result of inhibitory and cytotoxic effects on hematopoietic progenitor cells [43] and oxidative stress on bone marrow [44], limits the clinical application of 5-FU-based chemotherapies. In this research, SEP protected the immune system and hematopoietic function from injury caused by 5-FU, including up-regulating the indices of the spleen and thymus and reducing the atrophy of the spleen, thymus, and hematopoietic organs. It has been reported that 5-FU exerted cytotoxic effects through inhibiting thymidylate synthetase or incorporation into RNA and DNA and then promoting apoptosis by inducing ROS and activating caspase [45]. The result of AnnexinV-fluorescein (AV) and Propidium iodide (PI) double staining indicated that SEP decreased the apoptosis of both splenocytes and bone marrow cells *in vivo*, which were induced by 5-FU at each dose. Moreover, compared with the 5-FU alone treated group, the production of ROS was down-regulated in SEP and 5-FU combination treated groups in both splenocytes and bone marrow cells. It is well known that ROS forms as a natural byproduct of the normal metabolism of oxygen and plays important roles in cell signaling and homeostasis [46]. Moderate levels of ROS may function as signals to promote cell proliferation and survival, whereas severe elevation of ROS can damage cells and lipid membranes of subcellular components, resulting in lipid peroxidation, DNA damage and cellular apoptosis [47]. Therefore, SEP-suppressed excess ROS production induced by 5-FU is one of the mechanisms for reducing apoptosis in splenocytes and bone marrow cells. It has also been reported that the activation of caspase-3 is downstream of ROS in the apoptosis program and that the clearance of ROS inhibits the activation of caspase-3 [46, 48]. Caspase-3 is a member of the caspase (cysteine aspartic proteinases) family of enzymes, which, once activated, initiates the cell apoptosis program [49, 50]. The data obtained revealed that cleaved caspase-3 was up-regulated after 5-FU administration and down-regulated in the combination therapy group, indicating that SEP could suppress the activation of caspase-3 and then reverse the apoptosis caused by 5-FU in both spleen and bone marrow tissues. It is possible that SEP reversed 5-FU induced apoptosis by first suppressing ROS and then inhibiting caspase-3, thereby protecting the immune and hematopoietic systems from injury.

Collectively, our findings suggested that SEP and 5-FU coordinated to enhance the cytolytic activity of NK cells against tumor cells by stimulating the interaction between NKG2D and MICA *in vitro*. SEP augmented this interaction *in vivo* and reduced 5-FU-induced immunosuppression and hematopoietic injury by inhibiting ROS generation and caspase-3 activation. These results revealed the therapeutic potential of SEP and 5-FU in cancer treatment and further highlight the strategy of using an immunoregulatory agent in combination with a cytotoxic drug in the clinic.

## MATERIALS AND METHODS

### Antibodies and reagents

SEP (with a purity of 98.0%) was isolated as previously described [9, 10]. Briefly, crude, water-soluble polysaccharides from the eggs of sea urchins were separated and sequentially purified using Cellulose DE-52 and Sephacryl S-400 to yield SEP. High performance liquid chromatography (HPLC) (with a TSK gel 4000 PWXL column and a Waters 2414 refractive index detector (Sigma Aldrich, USA; mobile phase, 0.8 ml/min; column temperature, 30°C) was then used to assess the purity of SEP, and the profile revealed a single symmetrical sharp peak and a purity of 98.0%.

5-FU used in *ex vivo* experiments and in *ex vitro* experiments purchased from Dalian Meilun company (Dalian, Liaoning, China) and Jinyao company (Tianjin, China), respectively. Cell culture medium, including RPMI-1640, DMEM and α-MEM, and serum of fetal bovine or horse were bought from Gibco company (Vienna, NY, USA). Inositol, 2-mercaptoethanol, folic acid, 3-(4,5-Dimethylthiazol-2-yl)-2,5-di-phenyl tetrazolium bromide (MTT), 2′,7′-dichlorofluorescin diacetate (DCFH-DA), and Histopaque 1083 were obtained from Sigma-Aldrich (St. Louis, MO, USA). Recombinant IL-2 were purchased from R&D Systems Inc. (Minneapolis, MN, USA). Anti-CD34-FITC antibody, AnnexinV-fluorescein (AV) and the propidium iodide (PI) apoptosis detection kit were obtained from eBioscience (San Diego, CA, USA). The CytoTox 96 Non-Radioactive Cytotoxicity Assay kit was purchased from Promega (Madison, WI, USA). Primary antibodies against ADAM10 and DAP10 and were purchased from Abcam (Cambridge, MA, USA); cleaved caspase-3, total Akt (T-Akt), phosphorylated Akt (P-Akt), β-actin and anti-mouse/rabbit/goat IgG horseradish peroxidase (HRP)-linked antibodies were bought from SAB (College Park, MD, USA). Total Erk (T-Erk) and phosphorylated Erk (P-Erk) were obtained from Cell Signaling Technology (Beverly, MA, USA).

### Cell culture

The cell lines used in this study, including the human natural killer cell line NK-92, chronic myeloid leukemia cell line K562, human non-small cell lung cancer line A549, human hepatocellular carcinoma line HepG-2, mouse Lewis lung cancer (LLC) cell line and mouse hepatocellular carcinoma line H22, were purchased from ATCC (Manassas, Virginia, USA) and cultured in either RPMI-1640 medium or DMEM supplemented with 10% fetal bovine serum, 100 U/mL of penicillin, and 100 U/mL of streptomycin. NK-92 cells were maintained in α-MEM complete growth medium, which contained 0.2 mM inositol, 0.1 mM 2-mercaptoethanol, 0.02 mM folic acid, 200 U/mL recombinant IL-2, 12.5% horse serum and 12.5% fetal bovine serum. All of the cell lines were maintained at 37°C in a humidified atmosphere of 5% CO_2_ and used in the log phase of growth for all experiments.

### Animals

Male 6-week-old C57BL/6 mice and ICR mice were purchased from the Laboratory Animal Center of Yangzhou University (Yangzhou, Jiangsu, China). Animals were provided with continuous access to standard rodent chow and water and housed in a rodent facility at 22 ± 1°C with a 12 h light-dark cycle. All procedures were conducted in strict accordance with protocols approved by the Ethics Committee of China Pharmaceutical University.

### Killing activity analysis of NK cells

CytoTox 96 Non-Radioactive Cytotoxicity Assay was performed as described previously [51]. Briefly, target tumor cells were washed with PBS, resuspended with fresh NK-92 culture medium, and seeded in a 96-well plate at a density of 5 × 10^3^ cells/well. NK-92 cells were then added with relative effector to target (E:T) ratios of 10:1, 5:1, and 1:1. After the cells were incubated for 4 h at 37°C in a humidified atmosphere of 5% CO_2_, the supernatant in each well was harvested and measured by the CytoTox 96 Non-Radioactive Cytotoxicity Assay. The killing effect of NK cells against target cells was assessed with the following formula:

cytotoxicity = (experimental - effector spontaneous – target spontaneous)/(target maximum – target spontaneous) × 100%.

### Cell viability analyses

A549 and HepG-2 cells were harvested, washed with PBS and supplemented with RPMI-1640 or DMEM containing 10% fetal calf serum with a density of 5 × 10^4^ cells/mL. Then, 100 μl of cells were seeded in 96-well plates overnight. The cells were inoculated with 5-FU (with concentrations ranging from 1 to 50 μg/mL) for 24 h. Then, the cells were incubated with MTT solution (5 μg/mL) for 4 h. Next, the cells were lysed, and the purple formazan crystals were solubilized by dimethyl sulfoxide (DMSO) for detection at 490 nm with a microplate reader (Bio-Rad Model 6800, USA).

Cell viabilities were calculated using the following formula: cell viability (%) = (drug treatment – background)/(control – background) × 100%.

### Flow cytometry analyses

For analysis of MICA/MICB and NKG2D surface expression, tumor cells (A549 and HepG-2) or NK-92 cells were treated with 5-FU (with a concentration of 1, 10, or 20 μg/mL) or SEP (with a concentration of 50, 100, or 200 μg/mL), respectively. When the cells incubated were incubated with drugs for 12 or 24 h, they were collected and washed with PBS. Next, tumor cells were incubated with an anti-MICA/B-PE antibody (Miltenyi Biotech, Bergisch Gladbach, Germany), and the NK-92 cells were incubated with an anti-NKG2D-PE antibody (Miltenyi Biotech, Bergisch Gladbach, Germany). The resulting fluorescence was measured by flow cytometry analysis using a FACS flow cytometer.

### Measurements of soluble MICA (sMICA)

The effects of 5-FU on sMICA production in the supernatants of tumor cells were determined with a human sMICA ELISA kit (R&D Systems, MN, USA) according to the manufacturer's instructions. HepG-2 and A549 cells (1 × 10^4^ cells) were each placed in each well of a 96-well flat-bottomed microplate and exposed to different concentrations of 5-FU (ranging from 0.1 to 20 μg/mL) for 24 h. After incubation, the culture supernatants were harvested and tested for sMICA by ELISA.

### Western blot analyses

Cells were lysed in RIPA buffer in the presence of proteinase inhibitor cocktail (Generay, Shanghai, China). The protein concentration was determined using a BCA assay (Beyotime, Shanghai, China). Protein samples (20 or 40 μg) were separated on 12 or 15% SDS-PAGE (whichever was appropriate) and transferred to PVDF membranes. Then, the membranes were blocked in 1% BSA solutions for 1 h and incubated overnight with primary antibodies at 4°C. Next, the membranes were washed extensively with 0.1% Tween-20 in TBS (TBST) and incubated with secondary antibodies conjugated to horseradish peroxidase for 1 hour at 37°C. After washing with TBST again, the membranes were exposed using a Pierce(R) fast western blot kit chemiluminescence substrate. The blots were detected on a Bio-Imaging System using Quality One 1-D analysis software (Bio-Rad). All of the blots were stripped and reprobed using a monoclonal anti-β-actin antibody for determining if the proteins were loaded equally.

### Anti-NKG2D antibody blocking experiments

NK-92 cells were pretreated with either a NKG2D neutralizing antibody (R&D systems, MN, USA) or an isotype antibody (R&D systems, MN, USA) for 2 h. Then the cells were incubated in the presence or absence of SEP (200 μg/ml) for 24 h, collected, lysed and then analyzed using western blot assay.

### ADAM 10 plasmid construction and transfection

ADAM 10 overexpression plasmid was constructed by the following methods. The human ADAM10 overexpression plasmid and blank vector were purchased from Shanghai Jikai Gene company (Shanghai, China). The transfection of HepG-2 and A549 cells was then carried out with a Lipofectamine™ 3000 transfection reagent (Invitrogen, Carlsbad, CA, USA) with indicated ADAM10 overexpression plasmid. The cells were divided equally into three groups as follows: ADAM10 group (ADAM10-overexpressed plasmid group), vehicle group (blank-Vector-transfected group) and blank group (no DNA-transfected group). The efficiency of ADAM10 overexpression was evaluated by western-blot after cells were harvested 24 h post-transfection.

Further, the cells were treated with 5-FU (20 μg/mL) for another 24 h, harvested and stained with anti-MICA-PE antibody (R&D Systems, MN, USA) and analyzed using flow cytometry. In another experiment, the medium supernatant were collected and the sMICA production were determined with a human sMICA ELISA kit.

### H22- and LLC-bearing mouse models and administration

H22 ascites and LLC cells (0.1 ml, 5 × 10^5^ cells, > 98% viability) were transplanted subcutaneously into the right axilla of ICR or C57/BL6J mice. When the tumor grew to 100 mm^3^, the mice were randomly divided into seven groups with each group containing 10 mice. The mice were administered drug as follows: model group, normal saline; three 5-FU alone groups, 6.25, 12.5 or 25 mg/kg body weight of 5-FU; three 5-FU and SEP combination treatment groups, 6.25, 12.5 or 25 mg/kg body weight of 5-FU with 10 mg/kg body weight of SEP. All solutions were dissolved in normal saline, filtered through a 0.22 μm Millipore filter and administered every other day. H22-bearing ICR mice were administered by tail vein injection and LLC-bearing C57/BL6J mice were administered by intraperitoneal injection. 24 hours after the last drug administration, peripheral blood samples were taken and stored at 4°C for further use. Then, all animals were weighed and sacrificed *via* cervical dislocation. The spleen and thymus were immediately removed and weighed. Femurs of each group were taken and stored at 4°C for evaluation of hematopoietic function.

### Antitumor activity assay *in vivo*

The *in vivo* antitumor activity was expressed as a percent inhibitory rate, which was calculated as follows: [(A − B) / A] × 100%, where A and B are the average weights of the tumors from the control and experimental groups, respectively.

### Thymus and spleen indices and histochemical examinations

The thymus and spleen indices were calculated as thymus or spleen weight/body weight. The spleens, thymuses and femurs taken from all groups were fixed with 10% formalin and embedded with paraffin, and the sections (4 mm) were stained with hematoxylin and eosin (H&E). Histological changes were examined by light microscopy.

### Isolation of bone marrow mononuclear cells (BM-MNCs) and preparation of splenocytes

BM-MNCs were prepared as described previously [52]. Briefly, the femora and tibiae were harvested from mice in each group immediately after euthanization. BM cells were flushed into PBS containing 2% fetal calf serum using a 21-gauge and 5 ml syringe. Then, the cells were centrifuged through Histopaque 1083 to obtain BM-MNCs.

Splenic single cell suspensions were prepared as previous described [9].

### Measurements of leukocytes, erythrocytes, platelets, and CD34^+^ cells

Peripheral blood samples of each group were collected in test tubes with anticoagulants, and leukocytes, erythrocytes and platelets were counted with an automated chemical analyzer (COBAS INTEGRA 400 Plus, Roche, Switzerland).

Hematopoietic cells were collected from femurs by bone marrow aspiration, washed with PBS and resuspended in 100 μl of ice-cold PBS supplemented with 1% fetal calf serum, and then, anti-CD34-FITC monoclonal antibody was added to the suspension. The cells were incubated for 30 min at 4°C and then washed and resuspended in 500 μl of PBS. CD34^+^ cells were then detected by flow cytometry (BD LSR II Analyzer, BD Biosciences, USA).

### Flow cytometric detection of apoptotic cells

The apoptotic effects in splenocytes and BM cells in each group were detected using AV and PI apoptosis detection kits. Briefly, BM-MNCs or splenocytes were washed with PBS and suspended in 100 μl binding buffer; 5 μl of AV was added to the tube and incubated at room temperature in the dark for 15 min. After the cells were washed with 2 ml of binding buffer once and resuspended in 100 μl of binding buffer, 5 μl of PI was added. Next, the cells were analyzed immediately on a flow cytometer (BD Biosciences, San Jose, CA, USA). Signals of AV-FITV were measured at 488 nm for excitation and 525 nm for emission, and signals of PI were detected at 535 nm for excitation and 615 nm for emission.

The cell population fractions in different quadrants were analyzed using quadrant statistics. Early apoptotic cells and late apoptotic cells were shown in the lower right (LR) and upper right (UR) quadrants, respectively (13). The percentage of apoptotic cells was calculated as follows:

apoptosis rate (%) = (number of apoptotic cells) / (number of total cells observed) × 100%.

### Reactive oxygen species (ROS) assay

To determine the effects of ROS generation in splenocytes and BM-MNCs in each group, we carried out DCFH-DA assays to measure ROS generation. Briefly, BM-MNCs and splenocytes were washed and suspended in 100 μl of PBS with DCFH-DA for 40 min. Next, cells were collected and washed, and then the fluorescence intensity was measured at 488 nm for excitation and 525 nm for emission with a flow cytometer (BD Biosciences, San Jose, CA, USA).

### Statistical analyses

The data were analyzed using one-way analysis of variance (ANOVA) followed by Dunnett's test to identify any differences between either the control and combination treatment group or the drug alone-treated groups. The results are presented as the means ± standard deviations (SD). *P*-values of less than 0.05 were considered statistically significant.
